# *GmWRI1c* Increases Palmitic Acid Content to Regulate Seed Oil Content and Nodulation in Soybean (*Glycine max*)

**DOI:** 10.3390/ijms232213793

**Published:** 2022-11-09

**Authors:** Haowei Zheng, Duo Zhao, Wentao Shao, Yun Lu, Wenhui Wang, Yanjiao Hu, Jiajia Li, Shangshang Zhu, Xiaobo Wang

**Affiliations:** School of Agronomy, Anhui Agricultural University, Hefei 230036, China

**Keywords:** *GmWRI1c*, natural variation, nodule number, palmitic acid (16:0), seed oil content, soybean (*Glycine max*)

## Abstract

Soybean (*Glycine max*) is an important oil crop, but the regulatory mechanisms underlying seed oil accumulation remain unclear. We identified a member of the *GmWRI1s* transcription factor family, *GmWRI1c*, that is involved in regulating soybean oil content and nodulation. Overexpression of *GmWRI1c* in soybean hairy roots increased the expression of genes involved in glycolysis and de novo lipogenesis, the proportion of palmitic acid (16:0), and the number of root nodules. The effect of *GmWRI1c* in increasing the number of root nodules via regulating the proportion of palmitic acid was confirmed in a recombinant inbred line (RIL) population. *GmWRI1c* shows abundant sequence diversity and has likely undergone artificial selection during domestication. An association analysis revealed a correlation between seed oil content and five linked natural variations (Hap1/Hap2) in the *GmWRI1c* promoter region. Natural variations in the *GmWRI1c* promoter were strongly associated with the *GmWRI1c* transcript level, with higher *GmWRI1c* transcript levels in lines carrying *GmWRI1c*^Hap1^ than in those carrying *GmWRI1c*^Hap2^. The effects of *GmWRI1c* alleles on seed oil content were confirmed in natural and RIL populations. We identified a favourable *GmWRI1c* allele that can be used to breed new varieties with increased seed oil content and nodulation.

## 1. Introduction

Soybean is widely used in modern society. It provides health benefits for humans through its oil, protein, isoflavones, phospholipids, and vitamins [1,2,3]. With rapid population growth, there has been a significant increase in the demand for soybean oil. Therefore, it is of great significance to identify genes involved in the synthesis of oil, and in the regulation of this process, in order to breed new, high-quality soybean varieties.

Soybean oil consists of five main fatty acids; palmitic acid (16:0), stearic acid (18:0), oleic acid (18:1), linoleic acid (18:2), and linolenic acid (18:3). Palmitic acid and stearic acid are saturated fatty acids, while oleic acid, linoleic acid, and linolenic acid are unsaturated fatty acids [4]. Most studies on fatty acid components focus on food storage and safety. Saturated fatty acids can improve the oxidative stability of soybean oil, which is beneficial for storage. Unsaturated fatty acids can reduce the cholesterol content in human blood [5,6]. However, the roles of these five fatty acids in plant growth and development are unclear.

*WRI1* belongs to the ANT group of the AP2 transcription factor supergene family and promotes fatty acid synthesis by binding to the conserved AW-box in the promoters of its target genes [7,8]. *WRI1* was first discovered in Arabidopsis wrinkled seed coat mutants [9]. Previous studies have shown that *AtWRI1* and its homologs *BnWRI1*, *HaWRI1*, *CiWRI1*, and *GmWRI1a* can significantly increase the oil content in seeds of various species [10,11,12,13,14,15]; for example, *BnWRI1* regulates expression of glycolysis gene *BnBCCP* to increase seed oil content, and *GmWRI1a* regulates expression of de novo lipogenesis gene *GmACP* to increase seed oil content [11,12]. In soybean, *GmWRI1b* can improve plant structure and increase yield under field conditions [16]. A recent study proposed that *WRI1* is also involved in the regulation of soybean nodulation. In previous studies, the number of nodules in hairy roots was increased by the overexpression of *GmWRI1a/b* but decreased by the silencing of *GmWRI1a/b* [17]. These findings suggest that *WRI1* plays a vital role in plant growth and development. Thus, studies on soybean *WRI1* genes have important implications for crop health, growth, and yield.

The root nodule is a unique structure in legumes. Rhizobia fix nitrogen from the air into ammonia, thereby supplying nitrogen for the growth and development of their host plants. The roots and nodules of the host supply carbon and nutrients to rhizobia [18]. Symbiotic nodulation is a complex trait affected by nodulation factors, endogenous hormones, and the external environment. Previous studies have found that nodulation factors directly regulate nodulation in legume plants. Nodulation factors include nodule receptors (encoded by *NINs*), nodule factor receptors (encoded by *NFR1s*), symbiotic receptor kinases (encoded by *SYMRK/DMI2*), calmodulin kinases (encoded by *CCaMK/DMI3*), nodule signalling channel protein (encoded by *NSP*), and nodule marker oligopeptides (encoded by *ENOD40*) [19,20]. Plant hormones also play an important role in the nodulation process [21]. Nodulation is promoted by auxin and cytokinin [22,23,24] and inhibited by abscisic acid [25,26]. Legume nodulation is also affected by environmental factors. For example, light, nitrogen, and salt stress inhibit legume nodulation [27,28,29]. Recent studies have also shown that lipid metabolism genes are involved in regulating soybean nodulation [17,30,31], but it is still unclear how particular fatty acids affect the number of soybean nodules. Therefore, studies on the interaction between lipid metabolism and nodulation are of great practical significance.

*WRI1* encodes a transcription factor with critical biological functions in plants, such as regulating oil synthesis, seed development, nodulation, and yield [10,11,12,13,14,15,16]. However, only *GmWRI1a* and *GmWRI1b* have been studied in soybean so far. Exploring the biological functions of transcription factors encoded by other *GmWRI1s* and determining their selection patterns during domestication is very important for soybean breeding.

## 2. Results

### 2.1. Identification of WRI Gene Family Members in Soybean

WRI belongs to the AP2 transcription factor family. We downloaded the original HMM file of AP2 (PF00847) from the Pfam database, identified genes containing AP2 domains in the Williams 82 soybean reference genome, and then constructed a soybean-specific AP2 HMM. A total of 166 candidate genes containing AP2 domains were identified in the Williams 82 soybean reference genome using the AP2-specific HMM. The conserved structural domains of the candidate genes were further analysed using Pfam, NCBI-CDD, and Smart databases to remove genes lacking AP2 structural domains and those with severe deletions of AP2 structural domains. Finally, we identified 162 genes encoding AP2 transcription factors in soybean (Supplementary Dataset S2).

**Figure 1:**
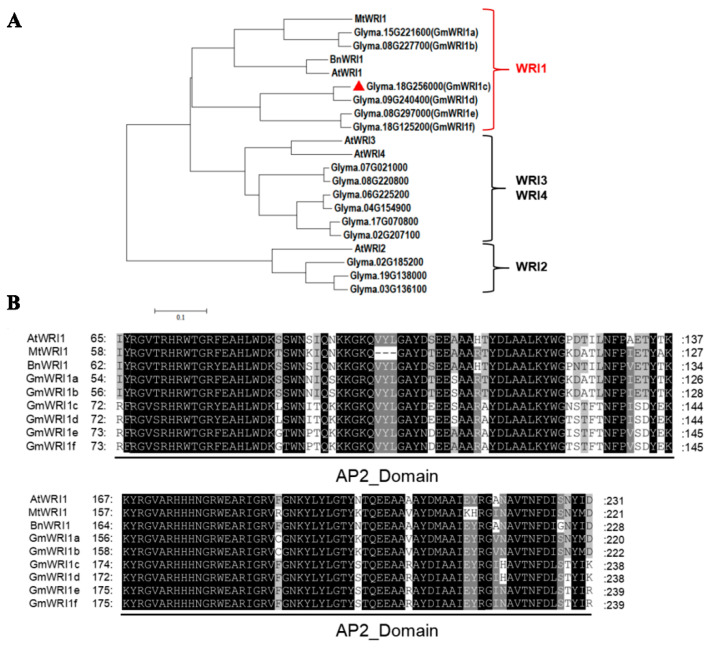
Phylogenetic and conserved domain analyses of GmWRI1s. (**A**) Phylogenetic tree of WRIs from *Arabidopsis*, soybean, *Medicago sativa*, and *Brassica napus*. (**B**) Conserved domain analysis of representative WRI1 proteins in *Arabidopsis*, soybean, *M. sativa*, and *B. napus*.

A phylogenetic tree was constructed with the neighbour-joining (NJ) method using the 162 soybean genes containing AP2 domains and *WRI* genes in *Arabidopsis* (*AtWRI1, AtWRI2, AtWRI3, AtWRI4)* (Supplementary Figure S1). Fifteen soybean genes with AP2 domains and Arabidopsis WRI genes were in the same evolutionary clade. An evolutionary analysis was conducted for these 15 soybean *WRI* genes and those from other species (Arabidopsis, M. sativa, and B. napus). Six soybean genes belonged to the *WRI1* group, three belonged to the *WRI2* group, and six belonged to the *WRI3* and *WRI4* groups (Figure 1A). The six *GmWRI1s* showed the highest homology with *AtWRI1*, and they all had two AP2 domains (Figure 1B). They were named *GmWRI1a*, *GmWRI1b*, *GmWRI1c*, *GmWRI1d*, *GmWRI1e*, and *GmWRI1f*. *GmWRI1a* and *GmWRI1b* have been reported to be involved in fatty acid synthesis and regulation of soybean nodulation [17].

The tissue expression patterns of the six *GmWRI1s* were determined on the basis of data from the Phytozome database. *GmWRI1a* and *GmWRI1*b were expressed in the root, nodule, and seed; *GmWRI1c* was expressed in the nodule; *GmWRI1d* and *GmWRI1f* were barely expressed in soybean; and *GmWRI1e* was expressed in the leaf and seed (Supplementary Figure S2). Searches of the Soybase database revealed that *GmWRI1c*, *GmWRI1d*, and *GmWRI1f* are located in soybean oil QTL intervals (*GmWRI1c* is located in the QTL intervals of seed oils 6–4 and seed oils 24–27; *GmWRI1d* is located in the QTL intervals of seed oils 4–11; and *GmWRI1f* is located in the QTL intervals of seed oils 27–10) [32,33,34,35]. As *GmWRI1d* and *GmWRI1f* are hardly expressed in soybean, we focused on *GmWRI1c.*

### 2.2. Conserved Function of GmWRI1c in Fatty Acid Synthesis

**Figure 2:**
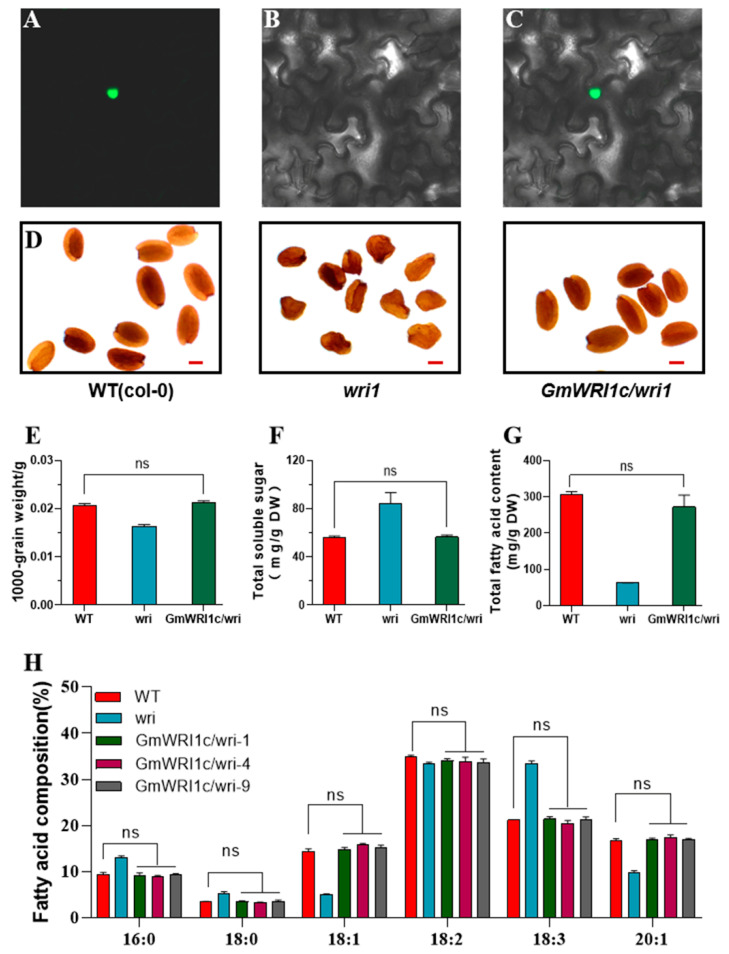
*GmWRI1c* restores the *Arabidopsis wri1* mutant phenotype. (**A**–**C**) Subcellular localisation of GmWRI1c. Fluorescence (**A**), bright-field (**B**), and merged (**C**) images of GmWRI1c-GFP. The magnification of microscope is 20 times. (**D**) Mature seeds of the wild type (Col-0), the *wri1* mutant, and *wri1* complemented with *35S::GmWRI1c/wri1*. Scale bar, 0.5 mm. (**E**–**H**) 1000-grain weight, total soluble sugar, total fatty acid content, and fatty acid composition of mature seeds of the wild type (Col-0), the wri1 mutant, and wri1 complemented with *35S::GmWRI1c/wri1*. *p*-values were determined by two-tailed two-sample Welch’s *t*-tests.

The *35S::GmWRI1c::GFP* fusion expression vector was transiently expressed in N. benthamiana leaves. The localisation analysis revealed that that *GmWRI1c* was located in the nucleus (Figure 2A–C), consistent with its role as a transcription factor.

We constructed a stably inherited *GmWRI1c* overexpression line in the Arabidopsis wri1 mutant background (Supplementary Figure S3). *GmWRI1c* completely restored the wrinkle phenotype, thousand-grain weight, soluble sugar content, total fatty acid content, and fatty acid composition of *wri1* mutant seeds (Figure 2D–H). These results indicate that *GmWRI1c* has a conserved function in fatty acid synthesis.

### 2.3. GmWRI1c Specifically Increases Palmitic Acid Content by Regulating Genes Related to Glycolysis and De Novo Lipogenesis

**Figure 3:**
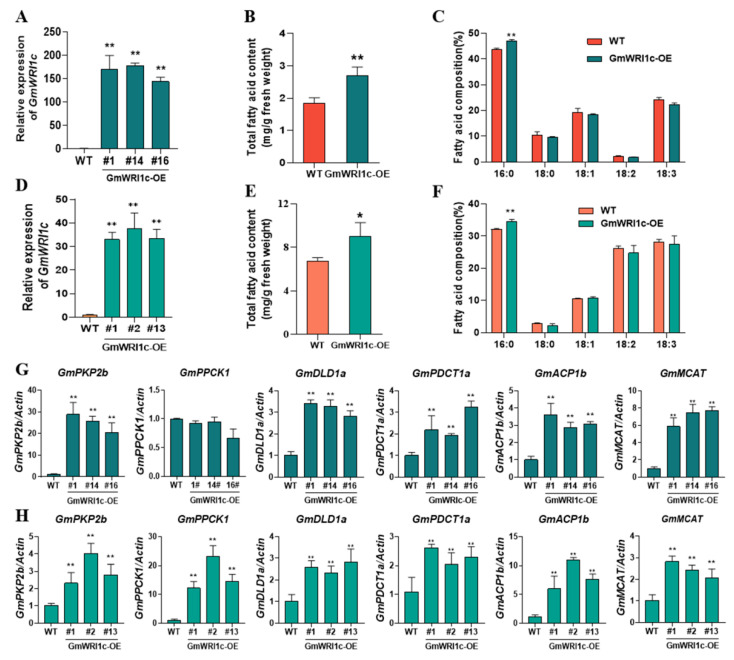
Analyses of GmWRI1c function in soybean. (**A**) Identification of transgenic hairy roots. (**B**,**C**) Total fatty acid content and fatty acid composition in *GmWRI1c*-OE transgenic hairy roots compared with control (values are means ± SD from three biological replicates). (**D**) Identification of transgenic root nodules. (**E**,**F**) Total fatty acid content and fatty acid composition in *GmWRI1c*-OE transgenic root nodules compared with control (values are means ± SD from three biological replicates). (**G**) Transcript levels of genes involved in glycolysis and de novo fatty acid synthesis in hairy roots of transgenic plants. (**H**) Transcript levels of genes involved in glycolysis and de novo fatty acid synthesis in root nodules of transgenic plants. * *p* < 0.05 and ** *p* < 0.01 (Student’s *t*-test).

To explore the function of *GmWRI1c* in soybean, we analysed lipids from both the roots and nodules of *GmWRI1c*-OE transgenic roots. Compared with the wild type, the line overexpressing *GmWRI1c* (Figure 3A,D) showed total fatty acid content in the roots and root nodules that was significantly increased by 38.22% and 32.8%, respectively (Figure 3B,E). Moreover, overexpression of *GmWRI1c* specifically increased the proportion of palmitic acid in roots and root nodules by 8.99% and 7.25%, respectively (Figure 3C,F). These results suggest that *GmWRI1c* promotes the biosynthesis of fatty acids, especially palmitic acid.

We then conducted qRT-PCR analyses to detect the transcript levels of genes involved in glycolysis and de novo lipogenesis in the *GmWRI1c*-OE transgenic hairy roots and nodules. Compared with the line expressing the empty vector, the *GmWRI1c*-OE line showed significantly (*p* < 0.01) increased transcript levels of genes involved in glycolysis (e.g., *GmPDHE*, *GmPKP*, *GmDLD*) and de novo lipogenesis (e.g., *GmPDCT*, *GmACP*, *GmMCAT*) (Figure 3G,H; Supplementary Figure S4). These results suggest that *GmWRI1c* specifically increases palmitic acid content by regulating genes involved in glycolysis and de novo lipogenesis.

### 2.4. Overexpression of GmWRI1c Promotes Nodulation Genes’ Expression and Increases Nodule Numbers in Soybean

**Figure 4:**
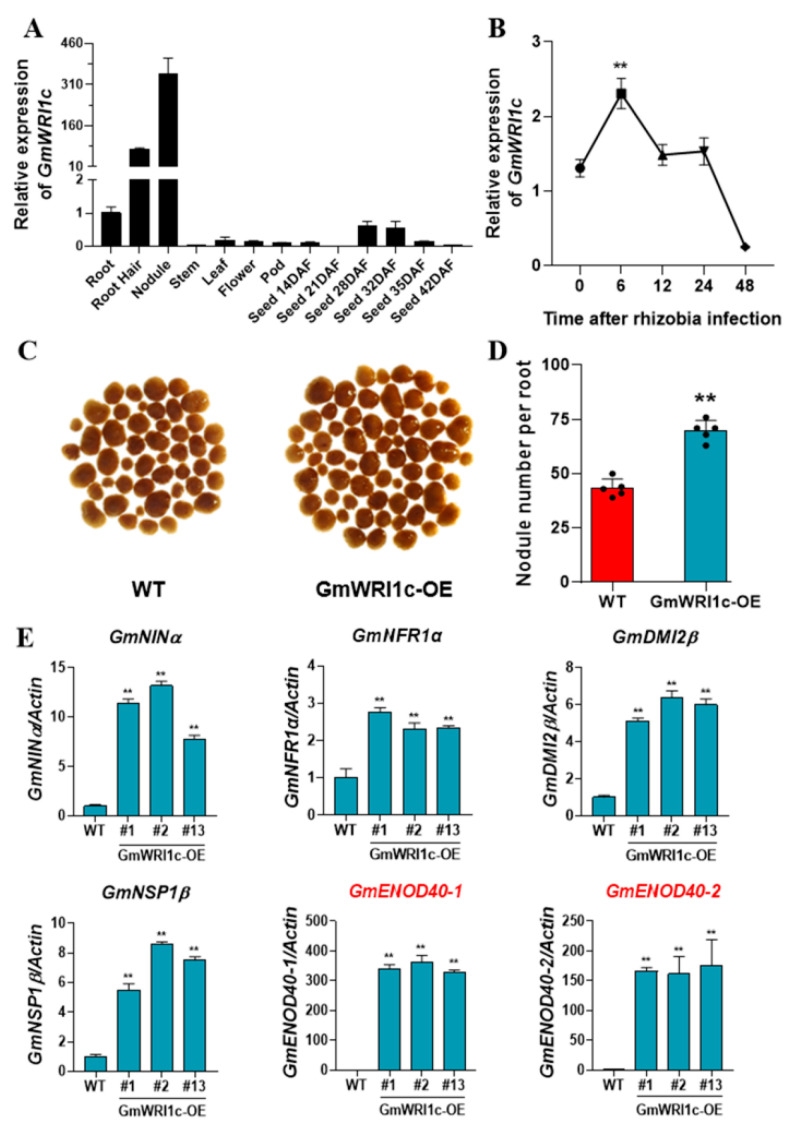
Effects of *GmWRI1c* on nodulation in soybean. (**A**) Transcript profiles of *GmWRI1c* in different tissues. (**B**) Transcript levels of *GmWRI1c* in roots after rhizobial infection. (**C**,**D**) Number of root nodules in *GmWRI1c*-OE transgenic hairy roots compared with control (*n* = 5). (**E**) Transcript level of gene encoding nodule factor in transgenic nodules. Values are means ± SD from three technical replicates. ** *p* < 0.01 (Student’s *t*-test).

The transcript levels of *GmWRI1c* in different soybean tissues were detected by qRT-PCR. The transcript levels of *GmWRI1c* were high in the root nodules and low in the seeds at 28 and 32 days after flowering (Figure 4A). Moreover, the transcript levels of *GmWRI1c* were significantly (*p* < 0.01) increased at 6 h after rhizobia infection (Figure 4B). Previous studies have shown that rhizobia invade root hair cells at 6–8 h after infection [36], so we hypothesised that *GmWRI1c* plays a role in the nodulation process of soybean. To test this hypothesis, we generated transgenic soybean hairy roots overexpressing *GmWRI1c* and then counted the number of root nodules. Compared with hairy roots expressing the empty vector, those of the *GmWRI1c*-OE line had significantly (*p* < 0.01) more root nodules. The average number of nodules was 69.8 on the transgenic roots compared with 43.4 on the roots of the control (Figure 4C,D), indicating that overexpression of *GmWRI1c* results in increased nodule numbers in soybean.

Previous studies have detected rapid up-regulation of early nodulation genes (e.g., *ENOD40*, *NIN*) in soybean roots after rhizobial infection [37,38]. *ENOD40* significantly influences nodulation and has been widely used as a marker gene for nodule formation [39,40]. For example, knocking down *ENOD40* in Lotus japonicus suppresses root nodule formation [41], and an AP2 transcription factor, *GmNNCI*, was found to suppress *GmENOD40-1* expression to regulate root nodule formation in soybean roots [42]. In this study, we also found that rhizobial infection induced *GmWRI1c* expression, so we speculated that *GmWRI1c* may regulate the expression of genes related to nodulation. To test this idea, we determined the transcript levels of nodulation-related genes in *GmWRI1c*-OE transgenic root nodules and found that *GmNIN, GmNFR1, GmDMI, GmNSP1*, and *GmENOD40* transcript levels were elevated. In particular, the transcript level of *GmENOD40* was increased by several hundred-fold (Figure 4E; Supplementary Figure S5). The increased transcript levels of nodulation-related genes in transgenic root nodules of *GmWRI1c*-OE coincided with increased numbers of root nodules.

### 2.5. Superior Allele of GmWRI1c^Hap1^ Increases Seed Oil Content and Nodule Numbers in Soybean

**Figure 5:**
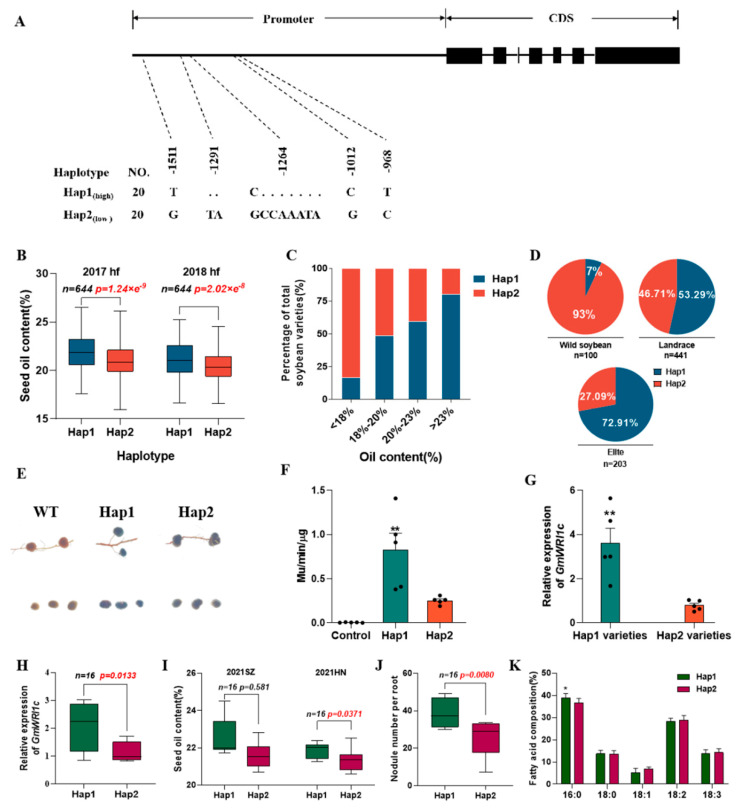
Effect of *GmWRI1c* on seed oil content and number of root nodules. (**A**) Haplotype analysis of *GmWRI1c* in 40 cultivated soybeans. (**B**) Boxplots of seed oil content for two haplotypes in 644 cultivated soybeans. (**C**) Percentages of two haplotypes of *GmWRI1c* in soybean lines grouped according to oil contents. (**D**) Distribution of two haplotypes of *GmWRI1c* in cultivated and wild soybeans. (**E**) GUS staining of transgenic nodules with gene expression driven by promoters of two *GmWRI1c* haplotypes. (**F**) Quantitative analysis of GUS activity in transgenic nodules (*n* = 5). (**G**) *GmWRI1c* transcript levels in root nodules of different soybean cultivars (*n* = 5). (**H**–**K**) Boxplots of gene transcript levels (**H**), seed oil contents (**I**), nodule numbers (**J**), and fatty acid composition (**K**) in soybean RILs with different haplotypes of *GmWRI1c*. 2017hf: grown in Hefei, China, in 2017. 2018hf: grown in Hefei, China, in 2018. 2021SZ: grown in Suzhou, China, in 2021. 2021HN: grown in Hainan, China, in 2021. Data are means ± SD from three biological replicates. *p*-values were determined with two-tailed two-sample Welch’s *t*-tests. * *p* < 0.05 and ** *p* < 0.01.

Sequence variations in *GmWRI1c* were investigated in 20 high-oil cultivated soybeans and 20 low-oil cultivated soybeans (Supplementary Dataset S3). For each sample, an approximately 4.0 kb region of *GmWRI1c* was sequenced, including its promoter, intron, and exon. In total, 99 single nucleotide polymorphisms (SNPs) and insertions and deletions (InDels) were detected in this 4.0 kb region (Supplementary Dataset S4). An association analysis using the mixed model method found that the *GmWRI1c* promoter had five polymorphic sites significantly (*p* < 0.01) associated with variations in seed oil content in two environments (Supplementary Table S2; Supplementary Dataset S5). We divided the five polymorphic sites into Hap1 and Hap2 (Figure 5A). A cleaved amplified polymorphic sequence (CAPS) marker (Supplementary Figure S6) was developed for one of the C/G mutations (chr18-54487077), and the genetic effects of the two haplotypes (Hap1/Hap2) were analysed. Analyses of 644 individuals in a natural soybean population revealed 383 carrying the Hap1 allele and 261 carrying the Hap2 allele (Supplementary Dataset S1). Correlation analyses were conducted between the two haplotypes of *GmWRI1c* and the oil content of soybean seeds in the two environments. The results showed that *GmWRI1c*^Hap1^ significantly (*p* < 0.01) increased the oil content of soybean seeds in both environments, increasing the seed oil content by 3.73% and 4.09%, respectively (Figure 5B). The 644 soybean lines were divided into four class intervals on the basis of their seed oil content (<18%, 18–20%, 20–23%, and >23%). The proportion of *GmWRI1c*^Hap1^ increased with increasing oil content. The proportion of *GmWRI1c*^Hap1^ was much higher than that of *GmWRI1c*^Hap2^ in the high-oil cultivated soybeans (Figure 5C). We investigated the frequency of the alleles and found that *GmWRI1c*^Hap1^ was present in 7%, 53.29%, and 72.91% of wild soybeans, landraces, and elite lines, respectively (Figure 5D). This result indicates that *GmWRI1c*^Hap1^ has undergone artificial selection during early soybean domestication. These results suggest that *GmWRI1c*^Hap1^ is a superior allele of *GmWRI1c* that improves soybean seed oil content.

To further understand the mechanism by which variations in the *GmWRI1c* promoter affect the oil content of soybean seeds, we analysed gene expression driven by the two haplotypes’ promoters (1600 bp upstream of the *GmWRI1c* transcription start site) in soybean hairy root transformation assays. The GUS activity was significantly higher (*p* < 0.01) in the transgenic nodules harbouring the *GmWRI1c*^Hap1^ promoter than in those harbouring the *GmWRI1c*^Hap2^ promoter (Figure 5E,F). Next, we determined the transcript levels of *GmWRI1c* in the nodules in soybean varieties with different haplotypes using qRT-PCR. The transcript levels of *GmWRI1c* were significantly (*p* < 0.01) higher in five high-oil cultivars with Hap1 than in five low-oil cultivars with Hap2 (Figure 5G; Supplementary Table S3). These data indicate that the differential expression of *GmWRI1c* in soybean nodules is the reason for differences in the oil contents of the soybean seeds.

To confirm the effect of the *GmWRI1c* allele type on seed oil content and the number of root nodules in soybean, the seed oil content, number of root nodules, and *GmWRI1c* sequences were analysed in a soybean RIL population (18 lines) derived from a cross between Jin dou 21 (JD21) and Fu shen dou (FSD). JD21 carries the Hap1 type of *GmWRI1c*, whereas FSD carries Hap2. Consistent with observations in natural populations, in the RIL population the transcript levels of *GmWRI1c* and the seed oil contents were higher in the lines with Hap1 than in those with Hap2 (Figure 5H,I). We also found that, compared with the RILs carrying Hap2, those carrying Hap1 had more root nodules (Figure 5J) and higher palmitic acid contents in the nodules (Figure 5K). These results confirm that the superior *GmWRI1c* allele is associated with higher seed oil content and a higher number of root nodules in soybean and, thus, will be useful for breeding new varieties with increased oil content.

## 3. Discussion

Since the discovery of *WRI1* in *Arabidopsis* wrinkled seed coat mutants, its homologs have been identified in diverse plant species [43]. Previous studies have found that *WRI1* can directly bind to the AW box in the promoters of genes involved in glycolysis and de novo lipogenesis to regulate their transcript levels [17]. In this study, we identified a WRI1 homolog that is expressed in root nodules and enhances the expression of genes related to glycolysis and de novo lipogenesis. This results in an increase in the proportion of palmitic acid out of total fatty acids. A recent study found that transient expression of *PaWRI1* in tobacco leaves specifically increased the proportion of palmitic acid [44], consistent with the results of our study. As palmitic acid is the main product of de novo lipogenesis [45,46], it is likely that *GmWRI1c* plays a main role in regulating de novo lipogenesis.

Symbiotic rhizobia–legume interactions are energy-demanding processes, and recent studies have shown that fatty acid composition and content strongly affect soybean nodule development and nodulation [17,30,31]. *GmWRI1a/b* directly regulates glycolysis and fatty acid biosynthesis, resulting in increased total fatty acid content and soybean nodulation [17]. Silencing of *GmACP* reduced the proportion of palmitic acid in transgenic hairy roots and inhibited soybean nodulation [30]. In the present study, overexpression of *GmWRI1c* significantly increased the transcript level of *GmACP*, leading to an increase in the ratio of palmitic acid in roots and nodules and more abundant root nodules. The results of our study and other studies suggest that palmitic acid has an important effect on soybean nodulation. Moreover, we detected a relationship between *GmWRI1c* alleles and the number of root nodules in the RIL population. The proportion of palmitic acid in the root nodules was higher in the RILs carrying the Hap1 type of *GmWRI1c* than in those carrying the Hap2 type. Therefore, we suggest that *GmWRI1c* may affect soybean nodulation by regulating the proportion of palmitic acid.

Legume domestication is one of the earliest technological innovations. From wild soybean to cultivated soybean, the seed size and seed oil content have undergone significant changes [47]. During soybean domestication, the seed size of *GmSWEET10a, POWR1, GmST105*, and *GmSS1* have significantly increased [48,49,50,51], and the seed oil content of *GmZF351, GmZF392*, and *GmST1* have significantly increased [52,53,54]. In this study, a nucleotide diversity analysis of *GmWRI1c* revealed a strong selective molecular footprint. *GmWRI1c*^Hap1^ was detected in 7%, 52.39%, and 72.91% of wild soybeans, landraces, and elite, respectively. The seed oil content was found to be significantly higher in lines carrying *GmWRI1c*^Hap1^ than in those carrying *GmWRI1c*^Hap2^ in both natural and RIL populations. These data indicate that *GmWRI1c*^Hap1^ is a beneficial haplotype that has been strongly favoured during soybean selection.

Recent studies have reported that divergence of gene expression driving trait evolution may be a common genetic mechanism in soybean domestication [55]. For example, natural variations in the promoters of *GmGBP* and *GsERD15B* affect gene expression, which alters the flowering time and salt tolerance in soybean [56,57]. The results of our transgenic experiments show that the promoter activity of *GmWRI1c*^Hap1^ is significantly higher than that of *GmWRI1c*^Hap2^. Further analyses of natural and RIL populations confirmed that *GmWRI1c*^Hap1^ is expressed at higher levels than is *GmWRI1c*^Hap2^. These data indicate that differential expression of *GmWRI1c* in soybean nodules is responsible for differences in seed oil contents among lines and varieties.

**Figure 6:**
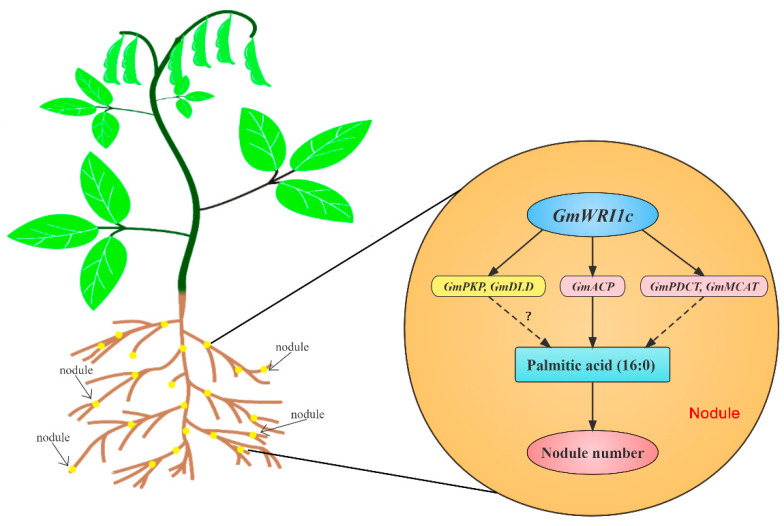
A working model for *GmWRI1c* regulation of soybean nodulation. ?—The mechanism of *GmPKP* and *GmDLD* increase palmitic acid is unclear.

In conclusion, we identified a crucial AP2 family gene, *GmWRI1c*, that is closely associated with nodule number and seed oil content in soybean. *GmWRI1c* promotes glycolysis and fatty acid biosynthesis and increases the proportion of palmitic acid to regulate soybean nodulation (Figure 6). Our analysis indicates that *GmWRI1c* has undergone artificial selection during early soybean domestication and identified a beneficial haplotype of *GmWRI1c*. The superior allele of *GmWRI1c*^Hap1^ is associated with higher seed oil content and an increased number of root nodules. Thus, the superior allele will be useful for breeding new high-oil soybean varieties.

## 4. Materials and Methods

### 4.1. Plant Materials and Growth Conditions

In total, 744 soybean germplasm resources were grown in Hefei, China. The experiment used a random block design, with 2 m long rows and 0.4 m between-row spacing. The population consisted of 100 wild soybeans, 441 landraces, and 203 elite lines from China and foreign soybean eco-regions (Supplementary Dataset S1). The Arabidopsis thaliana wri1 mutant (SALK_008559C) was used for transformation; all Arabidopsis and Nicotiana benthamiana (tobacco) plants were grown under standard growth conditions [58]. The transformation recipient, soybean variety Tianlong NO.1, and other soybean materials used for qRT-PCR were grown in a greenhouse under a 16 h/8 h (light/dark) photoperiod at 23/28 °C (night/day).

### 4.2. Identification and Bioinformatics Analysis of GmWRI1c

The latest reference genome of soybean and the hidden Markov model (HMM) profile of AP2 (PF00847) were downloaded from the Phytozome soybean genome and PFAM databases. The hmmsearch program in HMMER software [59] was used to search the AP2 HMM profile against the soybean genome, and the reliable results were screened based on an E-value less than or equal to 1 × 10^−10^. The soybean-specific AP2 HMM profile was constructed using the hmmbuild program and then the soybean reference genome was searched again with an E-value threshold 1 × 10^−10^. To avoid missing other members of the AP2 gene family, the AP2-type protein sequences from soybean reported on the NCBI website were used as query sequences for BLASTP alignment with the E-value threshold of 1 × 10^−5^. We combined the genes predicted using the two methods and removed the duplicates. If multiple transcripts corresponding to a single AP2 gene were obtained, only the most reliable one was retained. The protein sequences of the putative genes were submitted to NCBI–CDD3 and PFAM online databases for verification, and the putative genes without AP2 domains were excluded. The genes with an E value greater than or equal to 1 × 10^−20^ predicted by the Pfam database were also excluded. Finally, members of the soybean AP2 gene family were obtained with high confidence. For multiple alignment analysis, the amino acid sequences of WRI proteins from soybean, Arabidopsis, Medicago sativa, and Brassica napus were downloaded from the NCBI website. The phylogenetic tree was constructed with MEGA7.0 software using the NJ method [60]. The bootstrap tests were conducted with 1000 replicates, and other parameters were set to default.

### 4.3. Subcellular Localisation Analysis

The coding sequence (CDS) of *GmWRI1c* was amplified from the soybean cultivar Williams 82 using gene-specific primers (Supplementary Table S1). The CDS of *GmWRI1c* was cloned into pCAMBIA3301-GFP in-frame at the N-terminus of GFP using a one-step cloning kit (VAZYME, Nanjing, China). This construct was transferred into Agrobacterium tumefaciens GV3101, which was then injected into Nicotiana benthamiana leaves. After 36–48 h of incubation, the subcellular localization of *GmWRI1c* was observed in the epidermal cells of inoculated tobacco leaves under a laser scanning confocal microscope (Zeiss LSM880, Carl Zeiss, Jena, Germany) [61].

### 4.4. Generation of Transgenic Arabidopsis

The CDS of *GmWRI1c* was cloned into the pCAMBIA3301 vector harbouring the CaMV 35S promoter to generate *35S:GmWRI1c*. The empty vector served as the control. Subsequently, the vectors in the pCAMBIA3301 backbone were transferred into GV3101 and then used to transform Arabidopsis wri1 mutant plants using the floral-dip method [62].

### 4.5. RNA Extraction and cDNA Synthesis

Total RNA was isolated from soybean tissues using an RNA prep Pure Plant Kit (TaKaRa, Otsu, Japan), and first-strand cDNA was synthesised using a PrimeScript™ 1st Strand cDNA Synthesis Kit (TaKaRa).

### 4.6. Quantitative Real-Time PCR Analysis

Quantitative real-time PCR (qRT-PCR) analysis was performed with a CFX96TM Real-time System (BIO-RAD, Hercules, USA) with 10 μL of SYBR Mix (VAZYME, Nanjing, China), 6 μL of ddH_2_O, 1 μL of each primer, and 2 μL of cDNA template to a final volume of 20 μL. The implemented reaction procedure was 95 °C for 60 s, followed by 40 cycles of 95 °C for 30 s, 60 °C for 30 s, and 72 °C for 30 s. The relative gene expression levels were calculated using the 2^−ΔΔCT^ method [63] by normalization to GmACTIN (Glyma.19G147900^)^ [64] or AtACTIN7 (AT5G09810) in soybean and Arabidopsis, respectively [65]. All the primers used in this experiment are listed in Supplementary Table S1.

### 4.7. Soybean Hairy Root Transformation and Nodulation Assay

The CDS of *GmWRI1c* was cloned into the pCAMBIA3301 vector harbouring the CaMV 35S promoter to generate *35S:GmWRI1c*. The 1.6 kb promoter region of *GmWRI1c* was amplified from Tian Long No. 1 and Ji Lin Cai Li Xiang using specific primers (Supplementary Table S1). The promoter of *GmWRI1c* was cloned into the pCAMBIA1301 vector in-frame at the N-terminus of GUS using a one-step cloning kit (VAZYME). Subsequently, the freeze–thaw method was used to transform the pCAMBIA3301 vector containing *GmWRI1c*-CDS or pro-*GmWRI1c* into the soybean cultivar K599 (Tian Long No. 1) to obtain transgenic hairy roots according to methods published previously [66,67]. The transgenic soybean hairy roots and nodules were identified followed by the statistics for the transgenic hairy roots’ nodule numbers, and the RNA of transgenic roots and nodules were finally extracted.

### 4.8. Determination of Soluble Sugars Content, Total Fatty Acid Content, and Fatty Acid Composition

The soluble sugars content was determined using a plant soluble sugars content test kit, according to the manufacturer’s instructions (Jiancheng Bioengineering Institute, Nanjing, China). To determine total fatty acid content and fatty acid composition in soybean seeds, transgenic Arabidopsis seeds, soybean hairy roots and nodules, dry seeds (10 mg) of Arabidopsis, fresh soybean hairy roots (100 mg), and nodules (100 mg) were used to extract fatty acids. The fatty acid fraction from each sample was analysed by gas chromatography (SHIMADZU GC-2010 Plus, Japan) using heptadecanoic acid as an internal standard, according to the method described previously [68].

### 4.9. Determination of GUS Activity

Transgenic root nodules expressing two haplotypes of *GmWRI1c* and wild-type root nodules were stained with GUS staining buffer [69] containing 1 mM X-gluc (5-bromo-4-chloro-3-indolyl-β-D-glucuronide) (Gold BioTechnology, St. Louis, MO, USA), 100 mM sodium phosphate (pH 7.5), 0.5 mM potassium ferricyanide, 0.5 mM potassium ferrocyanide, 10 mM EDTA, and 0.1% (*v*/*v*) Triton X-100. The transgenic root nodules were incubated at 37 °C for 4 h and cleared with 70% (*v*/*v*) ethanol. For the quantitative assay, 100 mg transgenic fresh root nodules expressing each of the two haplotypes was ground in liquid nitrogen, and then GUS activity was detected according to the instructions of the GUS reporter quantitative assay kit (Coolaber, Beijing, China).

### 4.10. Soybean Oil Content Determination

The seed oil content of soybean grains was determined using an nIR grain quality analyser (DA7200, Perten Instruments, Springfield, IL, USA). The determination of seed oil content was conducted for each sample in triplicate.

### 4.11. Tassel Analysis and Genotyping

Association analysis was carried out between sites with genetic variations and oil content data using TASSEL 5.0 software. A haplotype analysis was performed to identify sites with significant effects on seed oil content (*p* < 0.05).

### 4.12. Statistical Analysis

All experimental data were obtained from three or more independent experiments with replicates and were analysed using Student’s *t*-test. Significant differences between the two datasets represented 95% confidence limits.

## Data Availability

All data supporting the findings of this study are available within the paper and within its supplementary data published online.

## Supplementary Materials

The following supporting information can be downloaded at: https://www.mdpi.com/article/10.3390/ijms232213793/s1.

## Abbreviations

Cleaved amplified polymorphic sequence	CAPS
Coding sequence	CDS
Haplotype	Hap
Hidden Markov model	HMM
Quantitative real-time PCR	qRT-PCR
Recombinant inbred line	RIL
