# Supplementary material for: GmWRI1c Increases Palmitic Acid Content to Regulate Seed Oil Content and Nodulation in Soybean (Glycine max)
Source: Int J Mol Sci. 2022 Nov 9;23(22):13793. doi: 10.3390/ijms232213793 (PMC9694093; doi:10.3390/ijms232213793)
Supplement: Supplementary file 1 [file ijms-23-13793-s001.zip › Supplementary Figures S1-S6+Tables S1-S3.pdf]

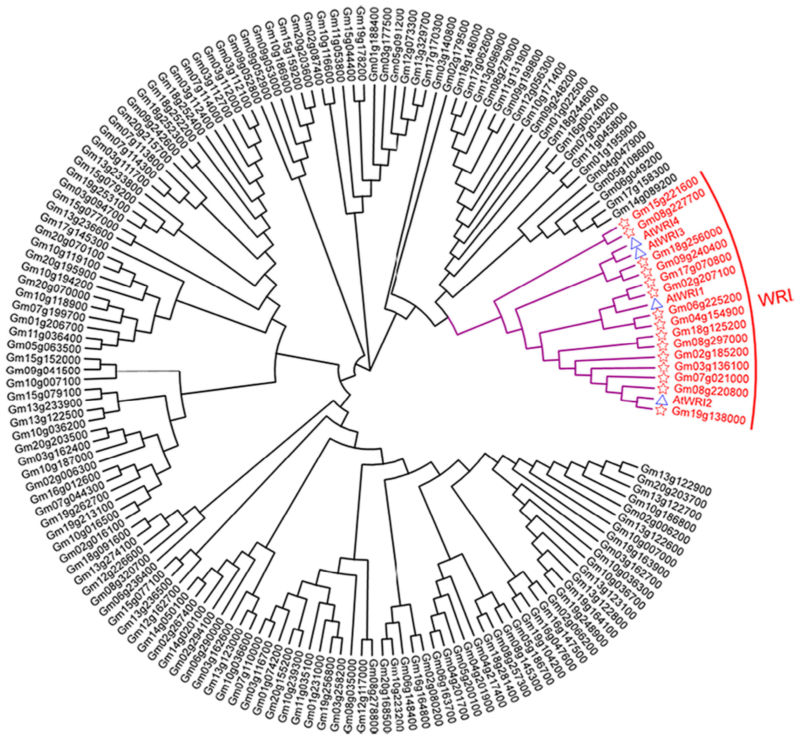

Figure S1. Unrooted phylogenetic tree of an AP2 transcription factor from Soybean. The alignment was generated using Clustal, and the unrooted phylogram was constructed with MEGA7 software using the neighbor-joining method.

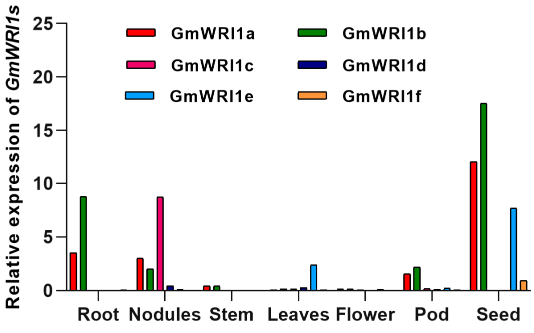

Figure S2. Expression patterns of *GmWRI1s* in different tissues of soybean plant. The public data are shown in phytozme (https://phytozome.jgi.doe.gov/).

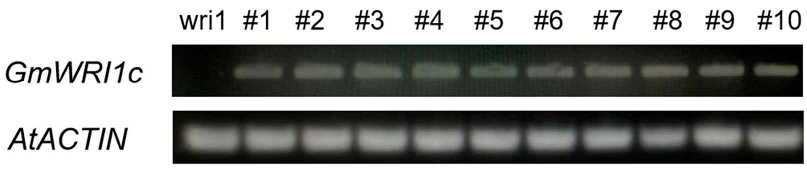

Figure S3. Identifying *atwri* transgenic plants of over-expressing *GmWRI1c*.

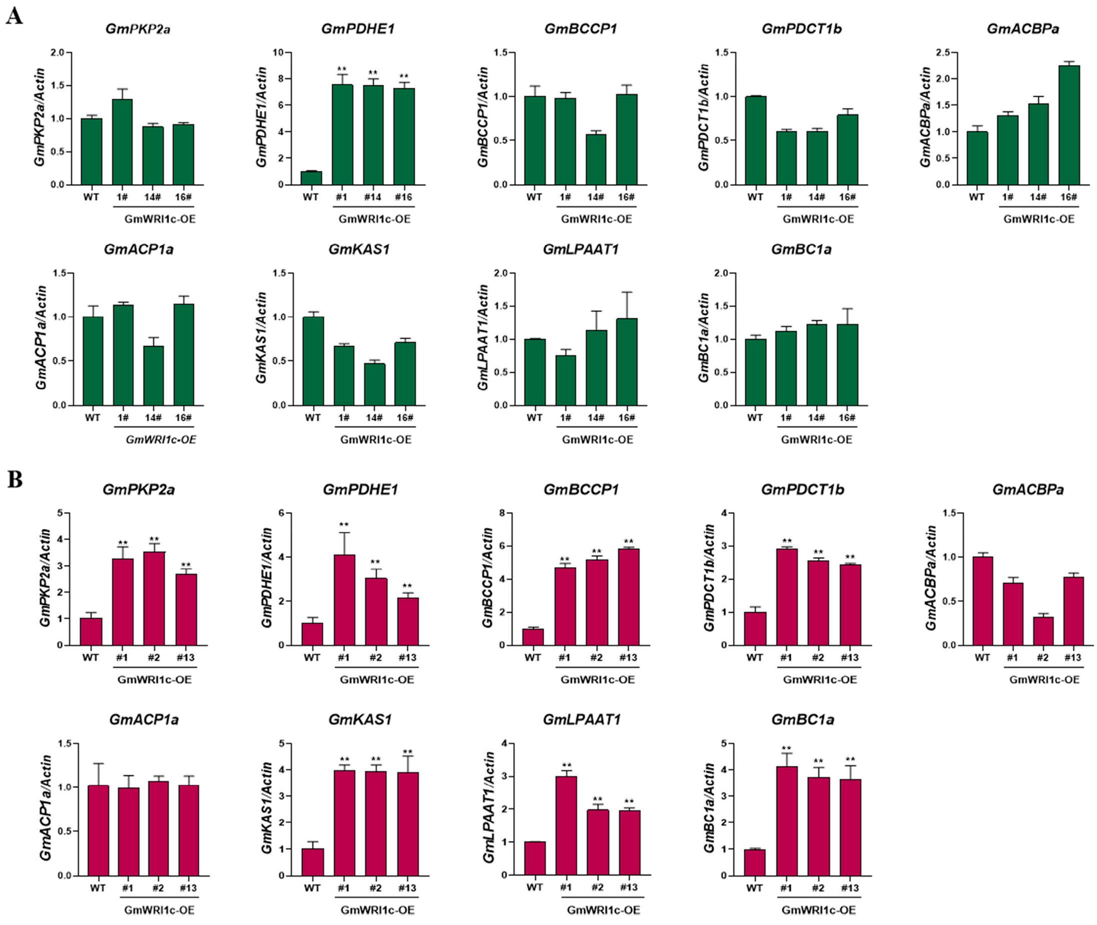

Figure S4. Effects of *GmWRI1c* on glycolysis, lipid metabolism genes expression in soybean.

(A) Expression levels of glycolysis and de novo fatty acid synthesis genes in hairy roots of transgenic plants. (B) Expression levels of glycolysis and de novo fatty acid synthesis genes in transgenic nodules. Expression levels were expressed as means ± SD from three technical replicates. $^{**}P < 0.01$ by Student's *t*-test labeled for significant difference.

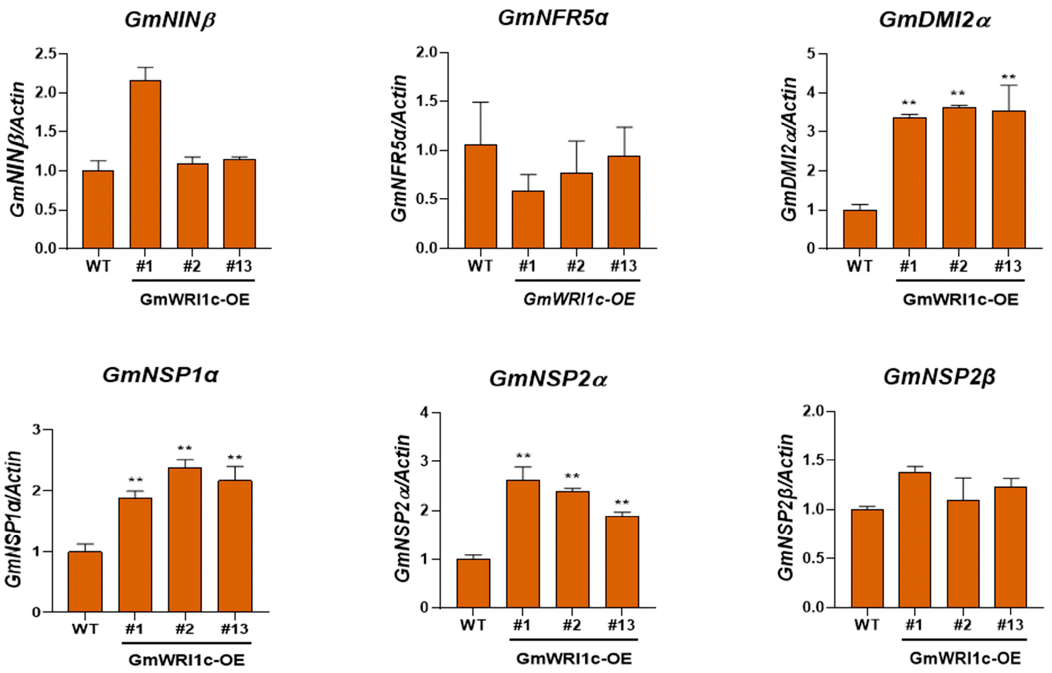

Figure S5. Effects of *GmWRI1c* on nodulation genes expression in soybean.

Expression levels were expressed as means ± SD from three technical replicates. **$P < 0.01$ by Student's t-test labeled for significant difference.

Figure S6

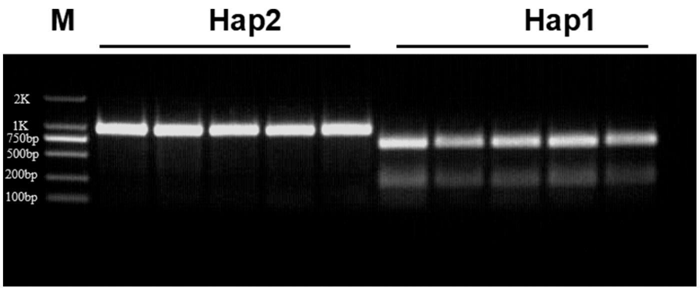

Figure S6. CAPS molecular markers for detection two haplotypes of *GmWRI1c*.

（M: DL 2000 Marker；1-5：Hap2 Soybean varieties；6-10：Hap1 Soybean varieties)

Table S1. Primers used in this study.

**Primers**	**Gene ID**	**primer sequence(5'--3')**
GmWRI1c(C/G)		AATACTCGGACACTCCCACCT<br>ATTCTAACCTCCCATACCCAT

GmWRI1c-cds		TCTAGAATGGTAATGATGAAAGAGAATATTATTG<br>GGATCCAAATATTGAGTGCAGGTCTCTTTCC
GmWRI1c-Pro		AGGCCTCCACCTTACCTATAACTATTTGC<br>CCTAGGTTCTTTTGTATTGTGATGTTCTAACTG
GmWRI1c-Genomic		GCAAATTATAAGAACAACACACACCTC<br>GGGATTGCTTTTTGTTAAGAAGAACTTC
LBb1.3		ATTTTGCCGATTTCGGAAC
LP（wri1）		AAAAACAGGCATCACCACAAC
RP（wri1）		TTTGTTCGGAGGACAAAGATG
qRT-GmActin	Glyma.19G147900	CTTCCCTCAGCACCTTCCAA<br>GGTCCAGCTTTCACACTCCAT
qRT-AtActin	AT5G09810	CCTCAGCACCTTCCAACAGATG<br>AACTCACCACCACGAACCAGAT
qRT-GmWRI1c	Glyma.18g256000	GAAGTTCTTCTTAACAAAAAGCAATCCCC<br>CAATGAAAAGCATGTATTGATGCAATTTAAC
qRT-GmACP1a	Glyma.13g214600	CCCTTTCAATTAAGGGAAGAAGT<br>CTGATTCATCTGGCAGTGCCAA
qRT-GmACP1b	Glyma.15g098500	TTCCCAATCTAGTTATGGTACCC<br>TCTTAACTATGTGGCACACCTTCT
qRT-GmPDCT1a	Glyma.08g213100	CGACGGCGCTAAAGGCGT<br>GCAGGAGTGCTTGGAAGAG
qRT-GmPDCT1b	Glyma.07g029800	CTATCCACAACCCACAGAGCAC<br>TCAGACTCTTGCCCTCTCACTC
qRT-GmPKP2a	Glyma.09g126300	TAGCTGCCTCACGATCCATTC<br>TGAAGGCCTCTGAGGCTGAG
qRT-GmPKP2b	Glyma.16g173100	ATGGCTCAGGTCGTGGCTT<br>TGTTCTCTCCCAATGGGAACA
qRT-GmPPCK1	Glyma.10g166600	TCTCCATAGTAATGGATCTCTGCC<br>GAAGTCCGCCAGTTTAAGATTGT

qRT-GmPDHE1	Glyma.05g141000	CGTCATTGTCTTCTTCCAACTC<br>TTCAGAACCTTGCTGCTTACC
qRT-GmDLD1a	Glyma.07g241600	CCCAGGACTTCCAAAATATTCAT<br>GAGCATAAGCATCTTTGACAGGC
qRT-GmBCCP1	Glyma.18g265300	AAGCACAACCAAAAGAGGCTG<br>GCATCAGCAGAAGTGCTTGG
qRT-GmBC1a	Glyma.05g221100	GTGCAAGTCCATTAGCTCGC<br>GCTAACTTGACCGACTTGCCTA
qRT-GmKAS1	Glyma.08g084300	CTTTCTCAGAGGAACGACGACC<br>CAGCTCCTCCCAAATATTCAGC
qRT-GmACBPa	Glyma.04g122900	TTATGGATTGTACAAGCAGGCC<br>AGTGATGTAATCACTCATTGCTTCG
qRT-GmLPAAT1	Glyma.17g131500	GTCCATGGATTCTATGGCCACT<br>CGACGACGGAGGAGTAGAGG
qRT-GmMCAT	Glyma.18g057700	GCTCTCAAGCACTCTCTCCCTC<br>CCCCAAAATGTCATTTGCCTT
qRT-GmNINα	Glyma.04g000600	TAACATGCGATGCTGATCTTG<br>TGATTTAGAGGCGAAGCTTGA
qRT-GmNINβ	Glyma.02g311000	CACCAACATCTTGAGTCTCTACC<br>TCTGCAAATCTCTGAATCCCCA
qRT-GmNFR1α	Glyma.02g270800	ATTCACGAGCACACTGTGCCT<br>CCAAAATCTGCAACCTTTCC
qRT-GmNFR5α	Glyma.01g179100	TTCCCTTTCTTCCTCTCCAC
qRT-GmDMI2α	Glyma.01g020100	GTCCTCAGTGGCCTTGACATT<br>ACACCCTTTTGCCTGCTTTG
qRT-GmDMI2β	Glyma.09g202300	CTGAGCGATTACAGTTCATGCA<br>TGCCTGCTTTGACAGTGCTATT
qRT-GmNSP1α	Glyma.16g008200	CAACACTTATCTTCTTCTCCAACT<br>GGAAGCATTTGCTATGTTGTTAGG

qRT-GmNSP1β	Glyma.07g039400	CACAACATCTATCATCTTCTCCC<br>GGAAGCATTTGCTATGTTGTTAGG
qRT-GmNSP2α	Glyma.06g110800	GTGACTTTGGTGGAGGAGGAG<br>CGAGTCAAAGACCGCTGAATA
qRT-GmNSP2β	Glyma.07g039400	AATCATTGCCAAGCGAAGCT<br>AGTCCAAAGCGAGGCAGAGA
qRT-GmENOD40-1	Glyma.01G028500	TCTCTCTTGAGTGGCAGAAGCA<br>TGGAGTCCATTGCCTTTTCG
qRT-GmENOD40-2	Glyma.02G036800	GAGTGGCGGAAGCAGATACAC<br>CTACATAGCCATAGAGACCCCAA

Table S2. Identified five polymorphic sites were associated with seed oil content.

Pos	polymorphic site	F		p	
		2017	2018	2017	2018
Chr-54486578	T/G	11.29070598	9.960466219	0.001784193	0.003125551
Chr-54486798	../TA	11.29070598	9.960466219	0.001784193	0.003125551
Chr-54486825	C......./GCCAAATA	11.29070598	9.960466219	0.001784193	0.003125551
Chr-54487077	C/G	11.29070598	9.960466219	0.001784193	0.003125551
Chr-54487121	T/C	11.29070598	9.960466219	0.001784193	0.003125551

Table S3. The seed oil content and relative expression of *GmWRI1c* in ten soybean accessions.

Variety code	Sample|type	Oil content-Avg(%)	Haplotype	Relative expression of *GmWRI1c* in nodules
JLCLX	Cultivar	17.24	Hap2	1.00
ZDD12330	Landrace	19.17	Hap2	1.04
ZDD15733	Landrace	19.14	Hap2	0.51
ZDD24126	Cultivar	17.92	Hap2	0.76

ZDD15808	Landrace	18.07	Hap2	0.63
ZDD24847	Cultivar	21.57	Hap1	1.68
L08Q104	Cultivar	25.37	Hap1	3.01
An1498	Cultivar	24.55	Hap1	5.65
WDD00596	Cultivar	23.78	Hap1	4.62
ZDD24601	Cultivar	24.88	Hap1	3.00
